# Detection of candidate gene *LsACOS5* and development of InDel marker for male sterility by ddRAD-seq and resequencing analysis in lettuce

**DOI:** 10.1038/s41598-022-11244-2

**Published:** 2022-05-05

**Authors:** Kousuke Seki

**Affiliations:** Nagano Vegetable and Ornamental Crops Experiment Station, Tokoo 1066-1, Souga, Shiojiri, Nagano, 399-6461 Japan

**Keywords:** Plant sciences, Plant breeding, Plant genetics, Plant reproduction

## Abstract

A new breeding method of F_1_ hybrid using male sterility would open an exciting frontier in lettuce breeding, a self-pollinating crop. Male sterility is a crucial trait in F_1_ hybrid breeding. It is essential to map the causative gene for using male sterility. The *ms-S*, male-sterile (MS) gene of ‘CGN17397’, was mapped to linkage group (LG) 8 by ddRAD-seq and narrowed down between two markers using two F_2_ populations. This region spans approximately 10.16 Mb, where 94 genes were annotated according to the lettuce reference genome sequence (version8 from ‘Salinas’). The whole-genome sequencing of the MS lines ‘CGN17397-MS’ and male-fertile (MF) lines ‘CGN17397-MF’ revealed that only one gene differed in the area of *Lsat_1_v5_gn_8_148221.1*, a homolog of *acyl-CoA synthetase5* (*ACOS5*), and was deleted in the MS lines. It was reported that *ACOS5* was needed for pollen wall formation and that the null mutants of *ACOS5* were entirely male sterility in some plants. Thus, I concluded that *Lsat_1_v5_gn_8_148221.1* designated as *LsACOS5* was a biologically plausible candidate gene for the *ms-S* locus. By using the structural polymorphism of *LsACOS5*, an InDel marker was developed to select the MS trait. The results obtained here provide valuable information for the genic male-sterility in lettuce.

## Introduction

Lettuce (*Lactuca sativa* L.), a cool-season vegetable crop, is stressed in high-temperature environments^1,2^. Increasing temperatures associated with climatic change have been shown to affect negatively the growth of lettuce, a major leafy vegetable, and necessitate the development of new cultivars with enhanced stress tolerance. Hybrids usually have better stress tolerance due to hybrid vigor than pure lines and have also been extensively used in leafy vegetable crops such as cabbage and Chinese cabbage to enhance crop production^3,4^. Harnessing hybrids are considered as one of the effective approaches for many leafy vegetable crops^5^, and the cultivation of F_1_ hybrids allows quantum jump in their productivity. Since a cultivation test has already confirmed that lettuce yield of F_1_ hybrids increased over the parent, and exploitation of hybrid vigor allowed to promise in improving the yield and other quality parameters^6^. Precise control over pollen fertility is a key factor in the production of F_1_ hybrids in self-pollinating crops^7^. Although the F_1_ hybrid breeding of the self-pollinating crops such as rice, soybean, wheat, and lettuce would challenge many common-sense assumptions in plant breeding, developments of hybrid rice using genic male sterility (GMS) and cytoplasmic male sterility (CMS) are already underway with great success in China^8,9^. In addition, numerous studies have been also performed for male sterility in soybean and wheat ^7,10–14^.

**Figure 1:**
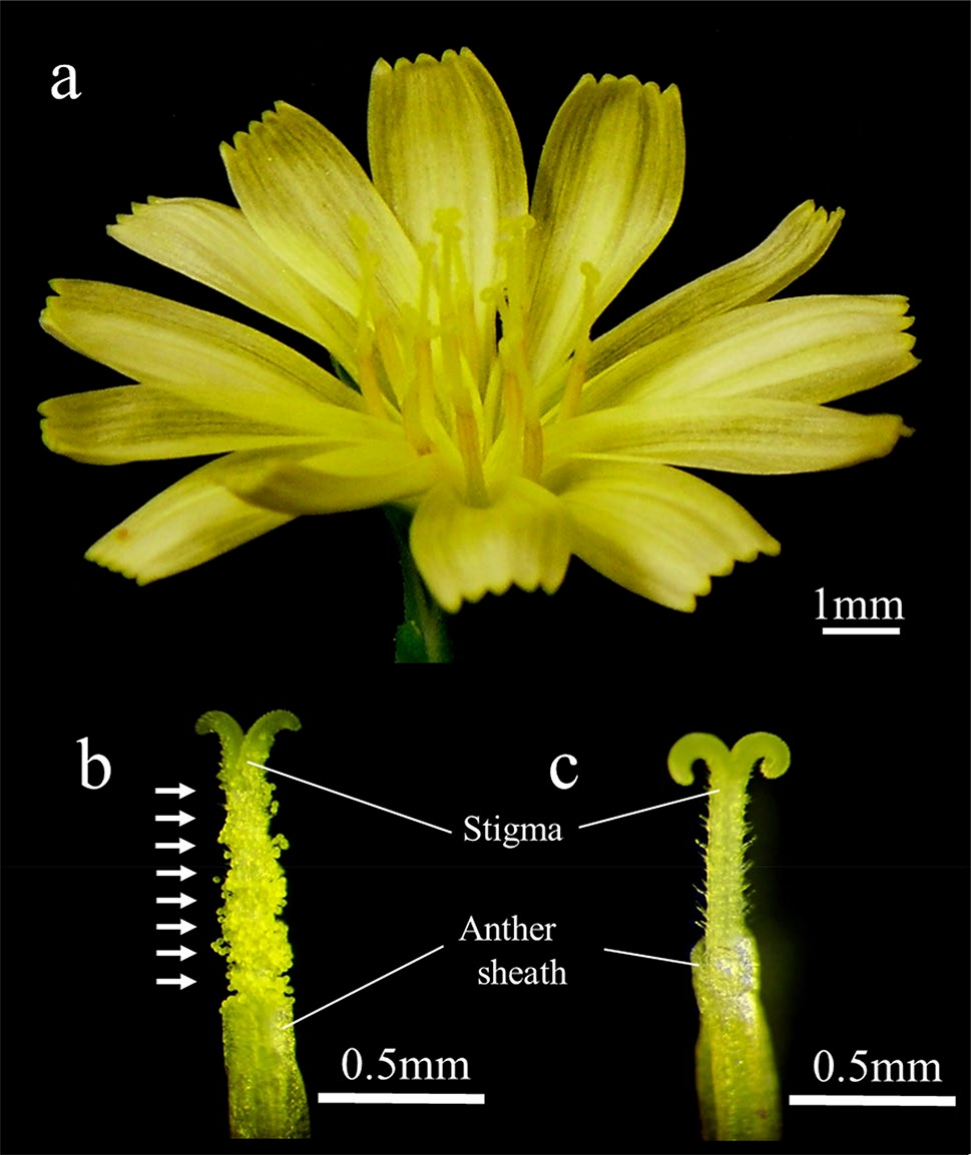
At the flowering time, stigmas emerge from the anther sheaths. (**a**) An inflorescence of *Lactuca sativa*: The inflorescence is composed of 7–15 yellow florets. (**b**) Pistil of a MF flower of ‘CGN17397-MF’: There are pollen grains on the stigma. White arrows indicate pollen grains. (**c**) Pistil of a MS flower of ‘CGN17397-MS’: There are no pollen grains on the stigma.

The present study began from the finding of a GMS plant in the inbred lines of ‘CGN17397’ (Fig. 1)^15^. Because lettuce has a compound autogamous floral structure, it is impossible to completely remove pollen from the flower^1^. Male sterility which can avoid unnecessary maternal self-pollination is not only an essential trait for the hybrid breeding approach in lettuce, and is also useful in the fundamental study of genetic and phenotypic investigations using F_1_ progeny such as disease resistance. In contrast to CMS, the phenotype of GMS is recognized after flowering. Hence, genetic markers linked to the male-sterile (MS) locus are needed to select MS plants at the pre-planting stage^16^. The markers for the *ms-S* gene have been developed by an amplified fragment length polymorphism (AFLP) technique so far, but all markers were located on the same side of the gene^15^. In this study, genetic mapping of the *ms-S* gene was conducted in two F_2_ populations obtained from a cross between MS and male-fertile (MF) plants. Additionally, by employing the whole-genome sequencing of MS lines ‘CGN17397-MS’ and MF lines ‘CGN17397-MF’, the candidate gene for male sterility was identified to develop a reliable PCR-based marker for MAS (Marker Assisted Selection).

## Results

### Inheritance of male sterility

MS phenotypes of the F_2_ individuals from a cross between MS plant ‘2008–83-MS’ and MF plant ‘UenoyamaMaruba’ and a cross between MS plant ‘CGN17397-MS’ and MF plant ‘Salinas’ were visually determined by whether there were pollen grains on stigmas or not at the flowering time. The MS trait derived from ‘CGN17397-MS’ was proposed to be controlled by a single recessive gene, according to the segregation of putative genotype of the male-sterile gene showing a 1:3 ratio in the two F_2_ populations (Table 1). These results are consistent with the previous study^15^.

**Table 1:** Segregation of male sterility in the two F_2_ populations.

Population	Maternal parent	Paternal parent	No. of sterile plants	No. of fertile plants	Total	Segregation ratio	*χ*^2^ (1:3)
F2	2008–83-MS	UenoyamaMaruba	22	68	90	1:3.09	0.90
F2	CGN17397-MS	Salinas	29	67	96	1:2.31	0.24

### Linkage analysis for male sterility trait by ddRAD-seq analysis

**Figure 2:**
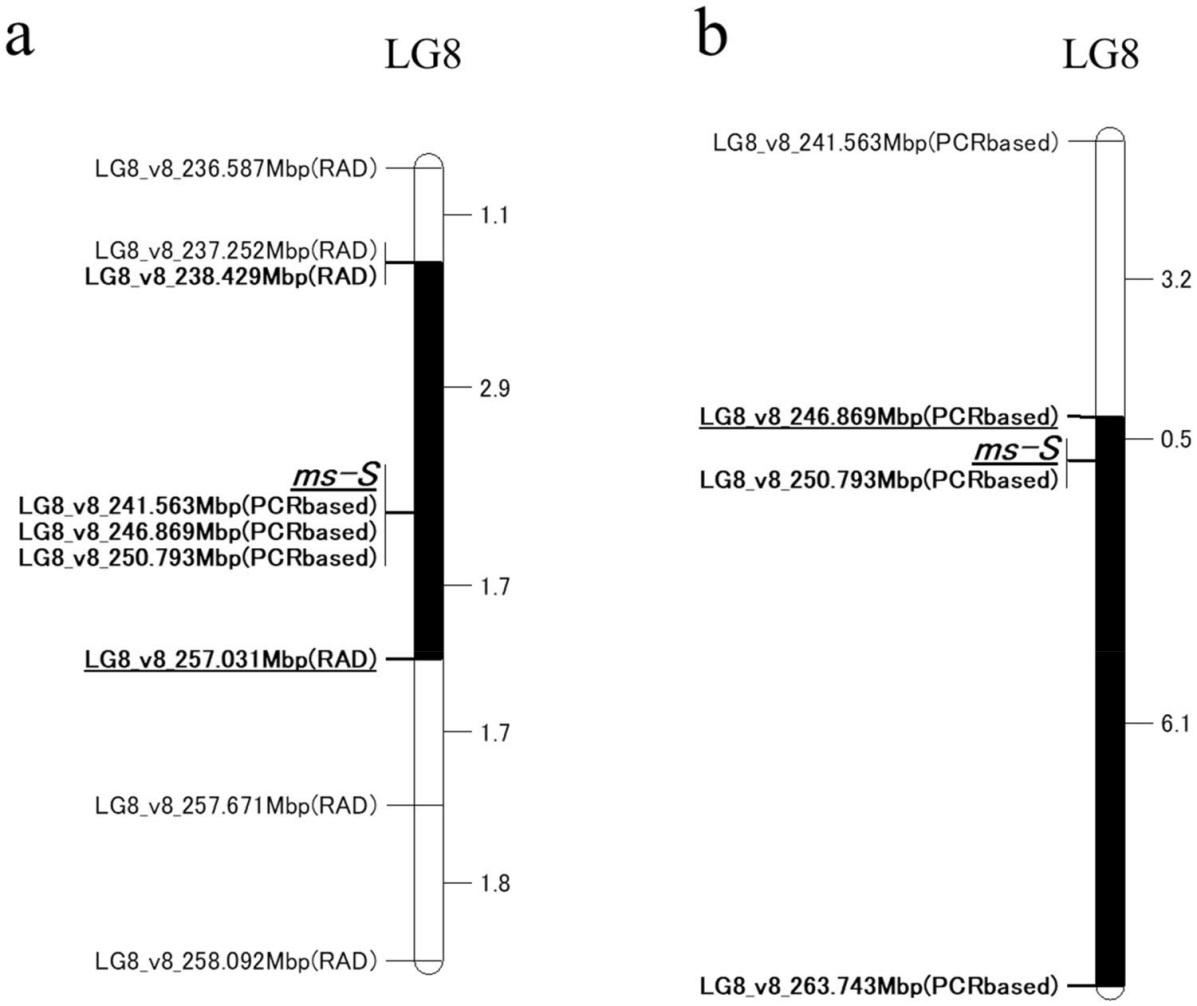
The mapped location of the *ms-S* locus on LG8 in two populations. Genetic distances (cM) were shown between the markers. “(RAD)” and “(PCRbased)” in the marker name indicate ddRAD-seq markers and PCR-based markers, respectively. “*ms-S*” indicates the position of the causal gene for male sterility. Black bars indicate *ms-S* locus. (**a**) Linkage mapping of the *ms-S* locus using an F_2_ population derived from a cross between ‘2008–83-MS’ and ‘UenoyamaMaruba’. (**b**) Mapping of the *ms-S* locus using an F_2_ population derived from a cross ‘CGN17397-MS’ and ‘Salinas’.

**Figure 3:**
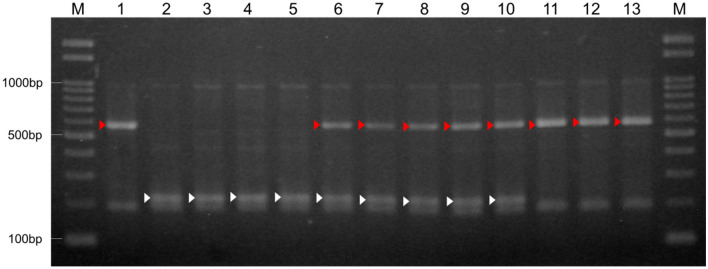
Agarose gel electrophoresis profiles for the Indel marker, *LG8_v8_250.793Mbp*, linked to the male sterility. Red arrows indicate the bands of 573 bp, and white arrows indicate the bands of 220 bp. *Lane 1* fertile parent ‘CGN17397-MF’; *lane 2* sterile parent ‘CGN17397-MS’; *lanes 3–5* sterile F_2_ plants, F_2_-2, F_2_-3, and F_2_-11; *lanes 6–9* fertile heterozygous F_2_ plants, F_2_-4, F_2_-8, F_2_-10, F_2_-13, and F_2_-14, *lanes 11–13* fertile homozygous F_2_ plants, F_2_-1, F_2_-5, and F_2_-6; *lane M* 100 bp ladder marker. Original gel is presented in Supplementary Figure S2.

For genetic mapping of the locus for the male sterility, double-digest restriction site-associated DNA sequencing (ddRAD-seq) analysis was conducted for constructing a linkage map using the F_2_ population from a cross between ‘2008–83-MS’ and ‘UenoyamaMaruba’. For the setting of RAD-R scripts^17^, BWA mode, construction method, and correction approach were “mem_60″, “ABH”, and ”6US” respectively. Then, the 1241 pairs of RAD tags in two parents were employed as codominant markers for genetic mapping of male sterility and used for linkage map construction (Fig. S1). By summarizing the linkage map, the total length of the linkage map was 1815.6 centi-Morgan (cM). Marker density ranged from 1.2 cM (LG2) to 2.0 cM (LG1) per marker. The number of markers in the linkage groups ranged from 93 (LG1) to 194 (LG5). Summary statistics of the linkage map are shown in Table 2. The segregation data of the genotype of the F_2_ population and the phenotype of MS traits showed that the *ms-S* gene was located at the position between 238.429 Mbp and 257.031 Mbp with the interval of 4.6 cM on LG8 (Fig. 2a). Genotyping using three PCR-based markers designed in this region was conducted for fine mapping (Table 3). However, the area could not be further narrowed in this population because these three markers showed complete cosegregation with male sterility (Fig. 2a). Then, the F_2_ population derived from a cross between ‘CGN17397-MS’ and ‘Salinas’ was employed to further mapping of the target locus using PCR-based markers. The gene of the male sterility was located at the position between 246.869 Mbp and 263.743 Mbp with the interval of 6.6 cM on LG8 (Fig. 2b), and *LG8_v8_250.793Mbp* indicated complete cosegregation with the male sterility based on the two F_2_ populations (Figs. 2, 3). The results of mapping using the two F_2_ populations demonstrated that the *ms-S* gene is located at the position between 246.869 Mbp and 257.031 Mbp on LG8 (Fig. 2).

**Table 2:** Summary of integrated lettuce linkage groups.

Linkage groups	Total mapped tags	Common tags		2008–83-MS unique tags		UenoyamaMaruba unique tags		Linkage construct marker		
								No. biallelic tags	Map length	Average interval between markers
		No.RAD-tags	(%)	No.RAD-tags	(%)	No.RAD-tags	(%)		(cM)	(cM)
LG1	124,945	22,471	18.0	54,780	43.8	47,694	38.2	93	184.5	2.0
LG2	129,078	21,774	16.9	57,304	44.4	50,000	38.7	145	180.7	1.2
LG3	156,126	29,616	19.0	69,645	44.6	56,865	36.4	153	203.6	1.3
LG4	233,307	40,703	17.4	105,380	45.2	87,224	37.4	180	255.3	1.4
LG5	206,664	36,684	17.8	93,595	45.3	76,385	37.0	194	273.5	1.4
LG6	123,784	20,567	16.6	55,848	45.1	47,369	38.3	121	173.2	1.4
LG7	124,880	21,275	17.0	53,338	42.7	50,267	40.3	106	161.6	1.5
LG8	183,044	33,217	18.1	82,951	45.3	66,876	36.5	134	221.1	1.6
LG9	131,647	21,519	16.3	59,308	45.1	50,820	38.6	115	162.0	1.4
Total	1,413,475	247,826	17.5	632,149	44.7	533,500	37.7	1241	1815.6	1.5

**Table 3:** Primers for the PCR-based markers in *ms-S* locus.

Primer name	Primer sequence (5'–3')	PCR product size (bp)			
		CGN17397-MS	Salinas	2008–83-MS	Uenoyama Maruba
LG8_v8_241.563Mbp_F	TTCGATCTCCGACGATTTATG	231	268	231	268
LG8_v8_241.563Mbp_R	CTAAGGAAACGGGAGGCAAT				
LG8_v8_246.869M_F	GTTTGGTTTGCGGATTCCTA	242	267	242	267
LG8_v8_246.869M_R	GTGCAACCAATTAGCATTCG				
LG8_v8_250.793Mbp_F	GATCCCTTCCAAAACTTGAGG	220	573	220	573
LG8_v8_250.793Mbp_MS_R	GGGCGGAGTCCATTATTTGT				
LG8_v8_250.793Mbp_MF_R	TGCTCAACGATCTTGTTTGTG				
LG8_v8_263.743M_F	TTTGAAAGCATAGGGATCATCT	297	304	304	297
LG8_v8_263.743M_R	GTTCATACCGTCGGATCGTT

### Identification of candidate genes in *ms-S* locus by whole-genome sequencing

**Figure 4:**
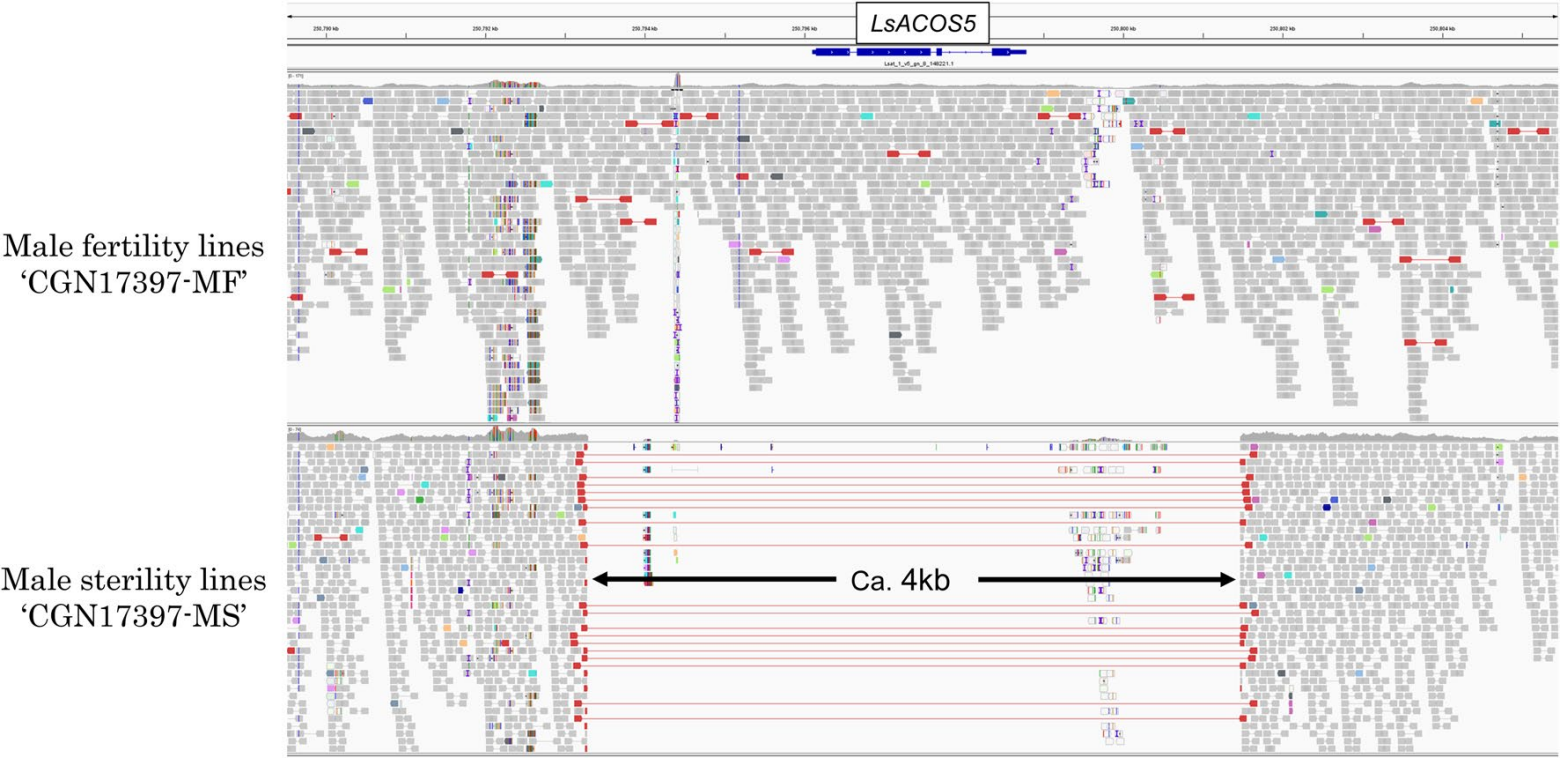
Screenshot of IGV software at around 250.7 Mb on LG8. Sequence reads of ‘CGN17397-MF’ and ‘CGN17397-MS’ aligned against a reference genome sequence. The deletion is displayed in only the MS lines ‘CGN17397-MS’.

**Figure 5:**
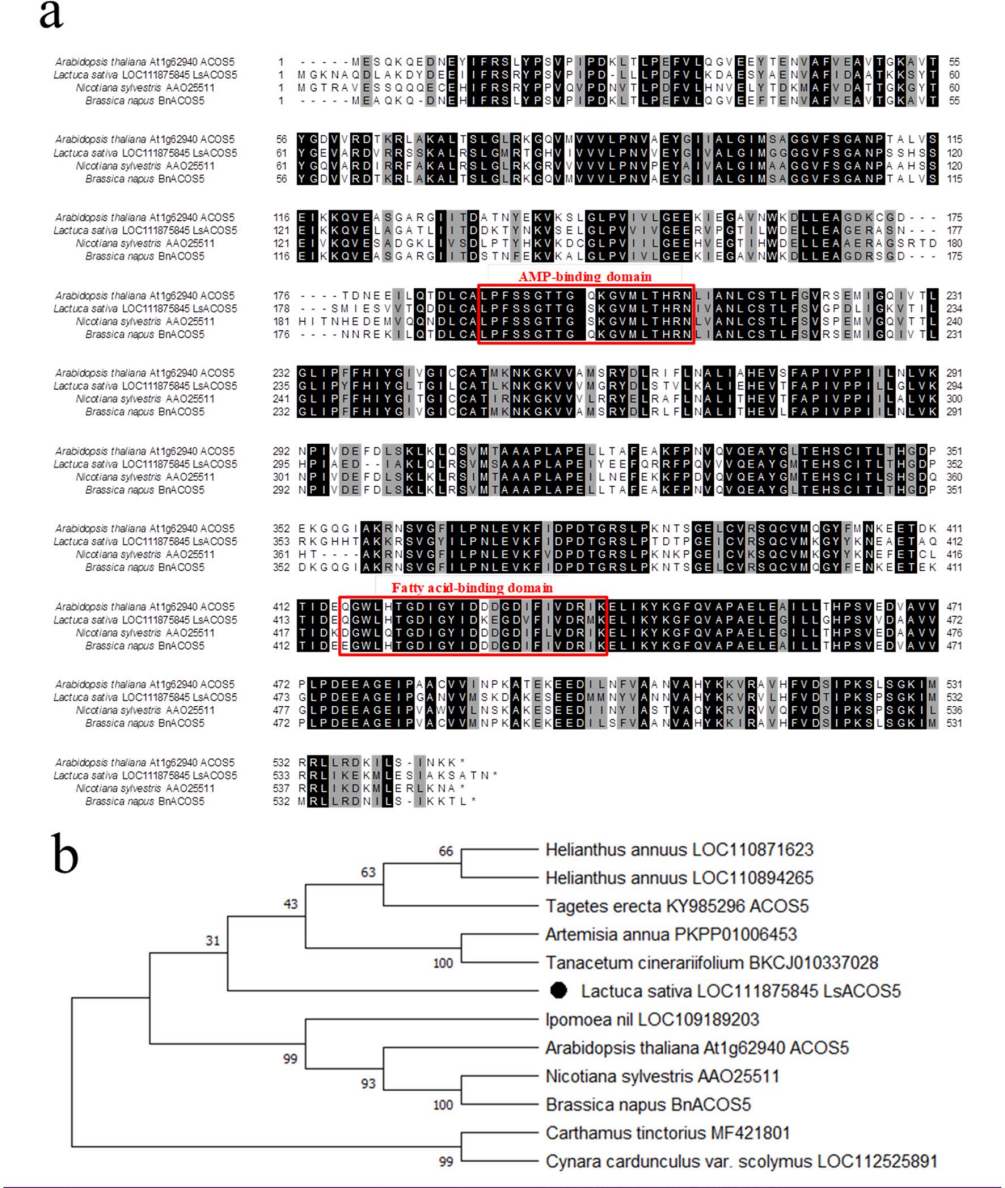
Sequence alignment of *LsACOS5* and its homologs. (**a**) Amino acid sequences alignment of *Arabidopsis thaliana* (*AT1G62940*), *Lactuca sativa* (*Lsat_1_v5_gn_8_148221.1*), *Nicotiana sylvestris* (*AAO25511*), and *Brassica napus* (*BnACOS5*). The sequences were aligned using ClustalW and displayed using BOXSHADE with MEGA X. Red frames indicate the conserved AMP-binding domain and fatty acid-binding domain. (**b**) A neighbor-joining phylogenetic tree of *LsACOS5* and its homologs in some plants. Bootstrap values are the percentage of 1000 replicates.

The *ms-S* locus was found to include 94 genes annotated according to the lettuce reference genome sequence (version8 from crisphead cultivar ‘Salinas’) (Table S1). Whole-genome sequencing data of the MS and MF lines revealed that a genomic region of about 4 kb containing the *Lsat_1_v5_gn_8_148221.1* was completely deleted in only the MS lines (Fig. 4). According to the reference genome sequence, *Lsat_1_v5_gn_8_148221.1* encodes an *acyl-CoA synthetase5* (*ACOS5*), which might be orthologous to *Arabidopsis* MS gene *AtACOS5*^18,19^. To further elucidate the relationship among *Lsat_1_v5_gn_8_148221.1*, *AtACOS5*, *AAO25511*, and *BnACOS5*, these four genes were examined for amino acid alignment by employing Clustal W. The results showed that there was significant conservation within the AMP-binding domain and the fatty acid-binding domain of *ACOS5*^20^ (Fig. 5a). The phylogenetic analysis showed that *Lsat_1_v5_gn_8_148221.1* was categorized into the *ACOS5* group, which is related to male sterility in some plant species^18,21^ (Fig. 5b). Based on the results, the gene might be the candidate gene for *ms-S* because of its homology with the known recessive MS gene and was designated as *LsACOS5*. For the other 93 genes, the genomic sequences were completely identical between the two lines (Table S1). And, some genes were reported to be expressed in flowers such as *SCD1* indicated as ORF 5^22^, but no genes were known to cause the null mutant to be the MS phenotype. The *LG8_v8_250.793Mbp* designed using the genomic regions of the candidate gene (Fig. 4, Table S1) had polymorphism between ‘CGN17397-MS’ and ‘CGN17397-MF’ and was completely cosegregated with the MS trait in the two F_2_ populations (Fig. 2). These results suggest that *LsACOS5* is a biologically plausible candidate gene for *ms-S*.

## Discussion

Because an F_1_ hybrid has a potential character that grows faster and has a shorter cultivation period in a field, the risk against bacterial disease accelerated by rain would be below. Thus, F_1_ hybrids are commonly anticipated to display high productivity under stressful conditions. In lettuce, the exploitation of the F_1_ hybrid could be one of the effective approaches to maintain a stable yield, particularly in tropical and subtropical regions. A new crisphead cultivar ‘Fine green’ was indeed the first F_1_ hybrid bred by Kaneko seeds CO., LTD. in Japan, but unfortunately, the technical detail of the breeding method was not announced publicly. In general, the MS plant is worth exploring as the key factor of F_1_ hybrid breeding, and several GMS mutants were also reported in lettuce so far^23^. The genetic mechanism is not understood, and this is the first report of the identification of the MS gene in lettuce. It is valuable to ascertain the genetic mechanism of MS plants to select a future breeding strategy.

In this study, the two F_2_ populations were used to locate the MS gene to the region between the two PCR-based markers, *LG8_v8_246.869Mbp* and *LG8_v8_257.031Mbp*. Although the genomic region of the *ms-S* locus was relatively large, the whole-genome sequencing for ‘CGN17397-MS’ and ‘CGN17397-MF’ revealed only 1 different gene, *Lsat_1_v5_gn_8_148221.1,* between 2 lines in these 94 annotated genes in the *ms-S* locus (Table S1, Fig. 4). The gene encoded an *acyl-CoA synthetase 5* (*ACOS5*) and was a potential ortholog of the key MS gene *ACOS5* in some plants such as *Arabidopsis*, Tobacco, and *Brassica napus*^24,25^ (Fig. 5a). The *ACOS5* acted as acyl-CoA synthase to regulate the biosynthesis of sporopollenin to affect male fertility, and a null mutant was entirely male-sterility^18^. ‘CGN17397-MS’ displayed normal vegetative growth and complete male-sterility insensitive to environmental conditions. There were no other obvious morphological differences between the MS and MF lines. Lettuce was generally only flowering for about two hours in the morning, but the MS lines could continue to flower through the afternoon. Thus, the MS mutants of lettuce and *Arabidopsis* showed phenotypic similarities^18^. I concluded that *LsACOS5* was a biologically plausible candidate gene for the *ms-S* locus (Figs. 2, 3, 4, Table S1).

In addition, the insertion/deletion (InDel) marker—*LG8_v8_250.793Mbp*—tightly linking to *LsACOS5* was developed. By using the InDel marker, it was possible to select MS plants for a conventional-breeding program (Figs. 1c, 3). Due to the structure of the lettuce flower, it was challenging to examine the inheritable characteristics of valuable traits^1^, such as disease resistance in only the F_1_ seeds because crosses produced not only F_1_ seeds but also self-pollinated seeds. Because only F_1_ hybrid seeds can be produced using GMS plants for crossbreeding, research on valuable traits that could not be analyzed in the past would be facilitated.

The F_1_ seed production system was needed to promote the commercial production of F_1_ hybrids. To propagate the F_1_ hybrid seeds in the case of rice, the maternal and paternal plants were alternately cultivated in a field to cross by the wind and artificial pollination^26^. But lettuce pollen was not dispersed by wind, the F_1_ seed production system has been already developed using insect pollination at a greenhouse. The fact that flies and bees were adopted for the system due to an absence of specialist pollinators of lettuce, the self-pollinating crop, could propagate the F_1_ hybrid seeds^27,28^. Moreover, the F_1_ hybrids are likely to be suitable for cultivation in not only fields but also plant factories. The trait of rapid growth was economically important for the cultivation in plant factories. The breeding of F_1_ hybrids suitable for cultivation in fields and plant factories is an issue for the future.

To date, genome editing technology makes it possible to create knockout mutants of the target gene. GMS plants generally have a problem of seed mixture for the MS and MF progeny. Still, a novel hybridization platform known as the third-generation breeding technique has been successfully selected for non-transgenic GMS seeds^8^. Combining these two techniques could also be applied for the F_1_ hybrid breeding in lettuce, and it converts any elite cultivars into a commercial MS plant and accelerates the development of F_1_ hybrid cultivars. The applications of the GMS plant initiative to the rise of considerable potential for lettuce breeding.

## Methods

### Plant materials

The plant materials were grown at the Nagano Vegetable and Ornamental Crops Experiment Station (Shiojiri City, Nagano prefecture, Japan; 36° 10′ N, 137° 93′ E). The genic MS plant was discovered as a spontaneous mutation in ‘CGN17397’ (Fig. 1). In this paper, the MS and MF lines were designated ‘CGN17397-MS’ (alias ‘MS1024’) and ‘CGN17397-MF’, respectively^15^. ‘CGN17397-MS’ and ‘CGN17397-MF’ were used for whole-genome sequencing. ‘2008–83-MS’ was obtained from a cross between ‘CGN17397-MS’ and a cultivar ‘Patriot’ at Nagano Vegetable and Ornamental Crops Experimental Station. A total of 90 individuals from the F_2_ progeny obtained from a cross between ‘2008–83-MS’ and ‘UenoyamaMaruba’ (*L. serriola*) were used for linkage analysis using ddRAD-seq. The MS trait was visually examined at the flowering time. Additionally, 96 individuals of F_2_ progeny obtained from a cross between ‘CGN17397-MS’ and ‘Salinas’ were used for further mapping using PCR-based markers.

### Linkage analysis based on ddRAD-seq

Genomic DNA was extracted from leaves using the Nucleo-Spin Plant II Extract Kit (Machery-Nagel, Duren, Germany). The RAD-seq library construction was performed following a previously described method^2,29^. The ddRAD-seq libraries were sequenced using the HiSeq4000 platform (Illumina, San Diego, CA, USA). Paired-end sequencing reads (100 bp × 2) were analyzed for ddRAD-seq tag extraction, counting, and linkage map construction using RAD-R scripts^17^. The read mapping was performed with the RAD tags in each parent against the lettuce reference genome sequence [version8 from crisphead cultivar ‘Salinas’ (https://genomevolution.org/coge/GenomeInfo.pl?gid=28333)]. The linkage map was graphically visualized using Mapchart and R/QTL^30,31^. Raw sequence data (FASTQ) in this ddRAD-seq were deposited in the DNA Data Bank of Japan (DDBJ) Sequence Read Archive (http://ddbj.nig.ac.jp/dra/index_e.html) under accession number DRA012711.

### Designing PCR-based markers and their amplification

Polymorphisms between parental lines around the *ms-S* locus, including insertion, deletion, and SNP, were surveyed to identify the marker sites using the IGV software^32^. Primers for amplifying the markers were designed using the Primer3 website (http://bioinfo.ut.ee/primer3-0.4.0/), and their IDs (names) were defined as (linkage group) _ (genome version) _ (genome position). PCR was conducted using 0.5 μL of DNA template, 0.4 μL of each primer (50 μM), 2 μL of dNTP (2 mM), 5 μL of 2 × PCR Buffer, 0.2 μL of KOD FX (1 U/μL, TOYOBO, Japan), and distilled water (dH_2_O) to a final volume of 10 μL. PCR conditions were as follows: at 94 °C for 5 min, 30 cycles of at 94 °C for 30 s, and at 61 °C for 30 s followed by 1 cycle at 72 °C for 4 min. 9 μL of PCR products were employed to electrophoresis on 2.5% agarose gel (Takara-bio, Japan) at 100 V after amplification.

### Resequencing analysis

Genomic DNA was extracted from young leaves of the two lines (‘CGN17397-MS’ and ‘CGN17397-MF’) using NucleoSpin Plant II (Machery-Nagel, Duren, Germany) and was used to construct paired-end sequencing libraries (100 bp × 2) and subjected to whole-genome sequencing using the HiSeqX (Illumina) and DNBSEQ-500 (MGI) platform. The resequencing analyses were conducted according to the previously described method^2^. Raw sequence data (fastq) for this resequencing analysis are available in the DDBJ Sequence Read Archive at accessions DRA012737.

### Phylogenetic analysis

The protein sequence of the candidate gene was searched for homologs from the plant species using basic local alignment search tools (BLAST) at the National Center for Biotechnology Information (http://www.ncbi.nlm.nih.gov/). Multiple sequence alignments of the full-length protein sequences were conducted using ClustalW and displayed using BOXSHADE (https://embnet.vital-it.ch/software/BOX_form.html). The phylogenetic tree was generated using MEGA X program^33^ using the neighbor-joining method with default parameters besides 1000 bootstrap replications.

### Ethical statement

The author assures that legislation on seed collection has been accomplished. Permission obtained from responsible authority to collect seeds.

### Ethical approval

All the experiments carried out on plants in this study were in compliance with relevant institutional, national, and international guidelines and legislation.

## Supplementary Information

Supplementary Information.
