# Supplementary material for: Detection of candidate gene LsACOS5 and development of InDel marker for male sterility by ddRAD-seq and resequencing analysis in lettuce
Source: Sci Rep. 2022 May 5;12:7370. doi: 10.1038/s41598-022-11244-2 (PMC9072324; doi:10.1038/s41598-022-11244-2)
Supplement: Supplementary file 1 — Supplementary Information. [file 41598_2022_11244_MOESM1_ESM.docx]

**Supplementary Information**

**Detection of candidate gene *LsACOS5* and development of InDel marker for male sterility by ddRAD-seq and resequencing analysis in lettuce**

**This file includes:**

Supplementary Fig. S1

Supplementary Fig. S2

Supplementary Table S1


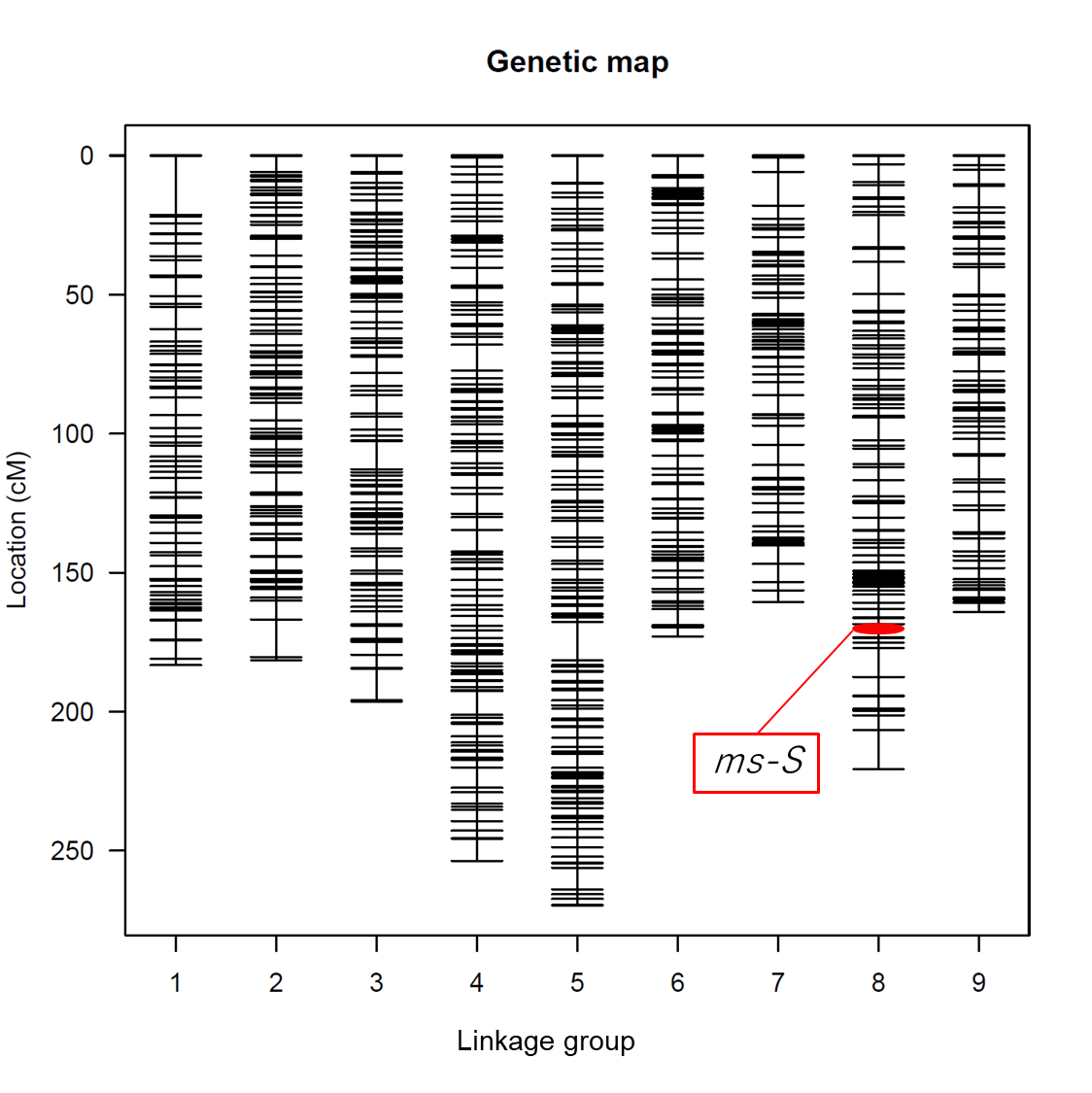


**Fig. S1**

Schematic representation of consensus map for an F_2_ population derived from a cross between ‘2008–83-MS’ and ‘UenoyamaMaruba’. The horizontal lines indicate the positions of loci of the markers on each linkage group.


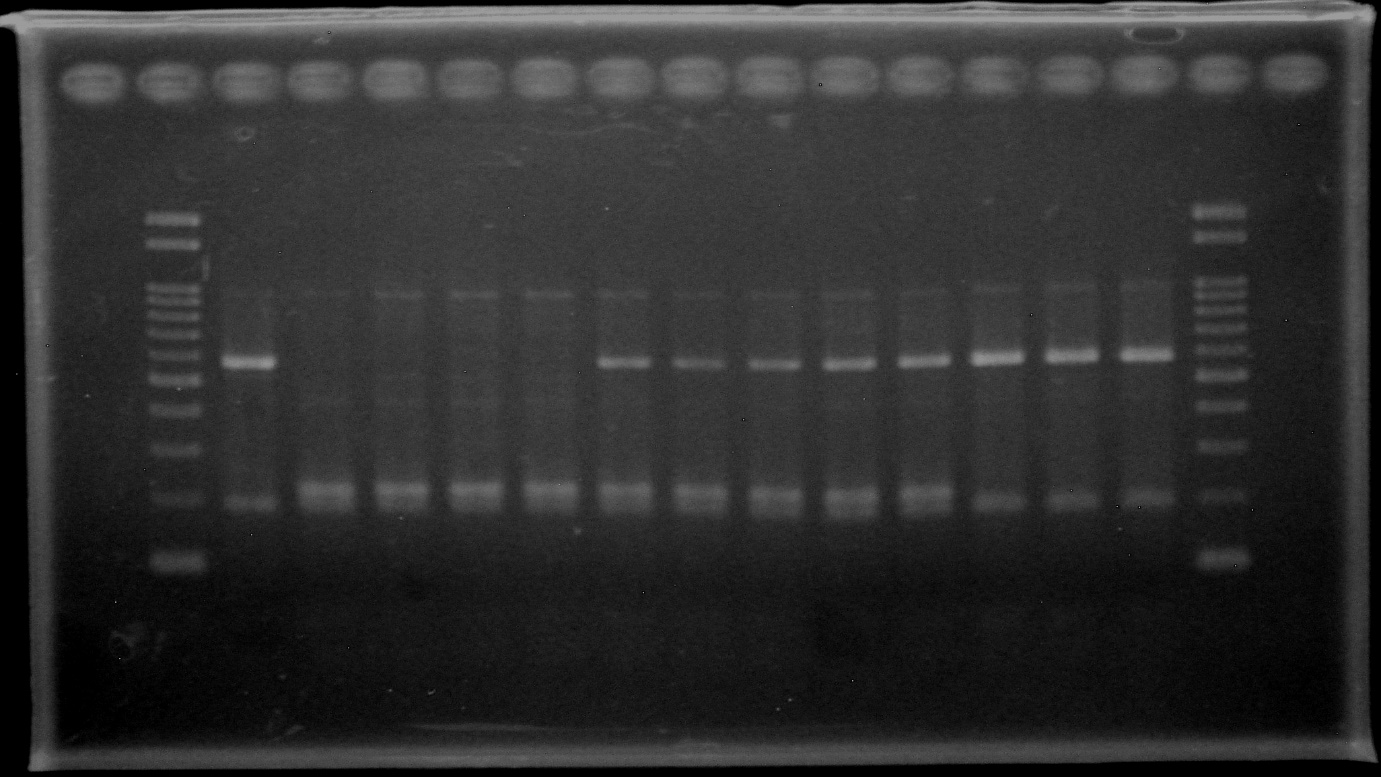


**Fig. S2**

Original image of agarose gel electrophoresis profiles for the Indel marker, *LG8_v8_250.793Mbp*, linked to the male sterility.

**Table S1**

Polymorphisms in the predicted genes between *LG8_v8_246.869Mbp* and *LG8_v8_257.031Mbp*. For 94 genes located on the *ms-S* locus, the genomic sequences were completely identical between ‘CGN17397-MS’ and ‘CGN17397-MF’ except for a genomic region of about 4 kb containing the *Lsat_1_v5_gn_8_148221.1*.

| ORF | Gene model name | Position of reference genome sequence | | Putative function | Sequencing |
| --- | --- | --- | --- | --- | --- |
|  |  | Start | End |  |  |
|  |  |  |  |  |  |
| 1 | Lsat_1_v5_gn_8_147101.1 | 246945395 | 246954005 | CCAAT-displacement protein, putative | Identical |
| 2 | Lsat_1_v5_gn_8_146520.1 | 247378637 | 247382754 | Expressed protein, putative | Identical |
| 3 | Lsat_1_v5_gn_8_146061.1 | 247611157 | 247611537 | Expressed protein, putative | Identical |
| 4 | Lsat_1_v5_gn_8_146081.1 | 247627209 | 247637718 | Expressed protein, putative | Identical |
| 5 | Lsat_1_v5_gn_8_146581.1 | 247675687 | 247694252 | Stomatal cytokinesis defective / SCD1 protein (SCD1) , putative | Identical |
| 6 | Lsat_1_v5_gn_8_146800.1 | 247953434 | 247954046 | Response regulator 2 , putative | Identical |
| 7 | Lsat_1_v5_gn_8_145740.1 | 248025320 | 248028304 | Expressed protein, putative | Identical |
| 8 | Lsat_1_v5_gn_8_147281.1 | 248152358 | 248153303 | Expressed protein, putative | Identical |
| 9 | Lsat_1_v5_gn_8_147260.1 | 248257431 | 248259544 | Exocyst subunit exo70 family protein C1 , putative | Identical |
| 10 | Lsat_1_v5_gn_8_147300.1 | 248413880 | 248416484 | Expressed protein, putative | Identical |
| 11 | Lsat_1_v5_gn_8_147201.1 | 248732092 | 248735156 | ACT domain repeat 4 , putative | Identical |
| 12 | Lsat_1_v5_gn_8_147341.1 | 248961954 | 248969589 | Expressed protein, putative | Identical |
| 13 | Lsat_1_v5_gn_8_147360.1 | 249102700 | 249106501 | Argonaute family protein , putative | Identical |
| 14 | Lsat_1_v5_gn_8_147381.1 | 249127002 | 249132963 | Cation-chloride co-transporter 1 , putative | Identical |
| 15 | Lsat_1_v5_gn_8_147540.1 | 249178886 | 249180334 | Expressed protein, putative | Identical |
| 16 | Lsat_1_v5_gn_8_147520.1 | 249270470 | 249274343 | Dihydrosphingosine phosphate lyase , putative | Identical |
| 17 | Lsat_1_v5_gn_8_148320.1 | 249418137 | 249418684 | Expressed protein, putative | Identical |
| 18 | Lsat_1_v5_gn_8_148361.1 | 249440320 | 249443316 | Alpha-L-arabinofuranosidase 1 , putative | Identical |
| 19 | Lsat_1_v5_gn_8_148400.1 | 249519260 | 249519777 | Expressed protein, putative | Identical |
| 20 | Lsat_1_v5_gn_8_148441.1 | 249629432 | 249630981 | HCP-like superfamily protein with MYND-type zinc finger , putative | Identical |
| 21 | Lsat_1_v5_gn_8_147461.1 | 249677669 | 249680672 | Rubber elongation factor protein (REF) , putative | Identical |
| 22 | Lsat_1_v5_gn_8_147900.1 | 250060257 | 250061238 | Cullin-associated and neddylation dissociated protein, putative | Identical |
| 23 | Lsat_1_v5_gn_8_148240.1 | 250265594 | 250266304 | ATP binding cassette subfamily B1 , putative | Identical |
| 24 | Lsat_1_v5_gn_8_147781.1 | 250529379 | 250530828 | WRKY DNA-binding protein 75 , putative | Identical |
| 25 | Lsat_1_v5_gn_8_147761.1 | 250595497 | 250599947 | Calmodulin-binding transcription activator protein with CG-1 and Ankyrin domains , putative | Identical |
| 26 | Lsat_1_v5_gn_8_147740.1 | 250600197 | 250605974 | Histidine kinase 3 , putative | Identical |
| 27 | Lsat_1_v5_gn_8_148201.1 | 250694004 | 250698546 | DNAJ heat shock N-terminal domain-containing protein , putative | Identical |
| 28 | Lsat_1_v5_gn_8_148221.1 | 250796099 | 250798785 | Acyl-CoA synthetase 5 , putative | Deletion in male sterility lines |
| 29 | Lsat_1_v5_gn_8_147801.1 | 250883703 | 250886810 | Ran BP2/NZF zinc finger-like superfamily protein , putative | Identical |
| 30 | Lsat_1_v5_gn_8_148461.1 | 250926740 | 250929158 | Glycosylphosphatidylinositol-anchored lipid protein transfer 1 , putative | Identical |
| 31 | Lsat_1_v5_gn_8_147881.1 | 251082074 | 251082769 | Villin 3 , putative | Identical |
| 32 | Lsat_1_v5_gn_8_147861.1 | 251209102 | 251212265 | Galactose oxidase/kelch repeat superfamily protein , putative | Identical |
| 33 | Lsat_1_v5_gn_8_148061.1 | 251452890 | 251453992 | Expressed protein, putative | Identical |
| 34 | Lsat_1_v5_gn_8_148040.1 | 251502891 | 251503221 | NAD(P)-binding Rossmann-fold superfamily protein , putative | Identical |
| 35 | Lsat_1_v5_gn_0_37180.1 | 251652368 | 251653508 | Expressed protein, putative | Identical |
| 36 | Lsat_1_v5_gn_8_147700.1 | 251858371 | 251864328 | Rubber elongation factor protein (REF) , putative | Identical |
| 37 | Lsat_1_v5_gn_8_147680.1 | 251946875 | 251951315 | DNA-binding HORMA family protein , putative | Identical |
| 38 | Lsat_1_v5_gn_8_147661.1 | 251969422 | 251969688 | Receptor for activated C kinase 1C , putative | Identical |
| 39 | Lsat_1_v5_gn_8_147640.1 | 252001329 | 252005010 | SCP1-like small phosphatase 5 , putative | Identical |
| 40 | Lsat_1_v5_gn_8_147620.1 | 252008686 | 252010173 | Expressed protein, putative | Identical |
| 41 | Lsat_1_v5_gn_8_147560.1 | 252144648 | 252146886 | ELMO/CED-12 family protein , putative | Identical |
| 42 | Lsat_1_v5_gn_8_148781.1 | 252307245 | 252312025 | Transcription regulator protein, putative | Identical |
| 43 | Lsat_1_v5_gn_8_148840.1 | 252830254 | 252831365 | Xyloglucan endotransglucosylase/hydrolase 5 , putative | Identical |
| 44 | Lsat_1_v5_gn_8_148880.1 | 252865566 | 252866140 | PPPDE putative thiol peptidase family protein , putative | Identical |
| 45 | Lsat_1_v5_gn_8_148901.1 | 252867010 | 252869686 | Exostosin family protein , putative | Identical |
| 46 | Lsat_1_v5_gn_8_149060.1 | 252963951 | 252964484 | Expressed protein, putative | Identical |
| 47 | Lsat_1_v5_gn_8_149401.1 | 253219678 | 253227404 | Zn-dependent exopeptidases superfamily protein , putative | Identical |
| 48 | Lsat_1_v5_gn_8_149361.1 | 253270230 | 253272069 | Microsomal signal peptidase 12 kDa subunit (SPC12) , putative | Identical |
| 49 | Lsat_1_v5_gn_8_149340.1 | 253276572 | 253278836 | Minichromosome maintenance (MCM2/3/5) family protein , putative | Identical |
| 50 | Lsat_1_v5_gn_8_149320.1 | 253345360 | 253346445 | Homolog of yeast oxidase assembly 1 (OXA1) , putative | Identical |
| 51 | Lsat_1_v5_gn_8_149261.1 | 253549014 | 253550754 | Galactose oxidase/kelch repeat superfamily protein , putative | Identical |
| 52 | Lsat_1_v5_gn_8_149240.1 | 253578298 | 253578519 | Expressed protein, putative | Identical |
| 53 | Lsat_1_v5_gn_8_149221.1 | 253584013 | 253586316 | Expressed protein, putative | Identical |
| 54 | Lsat_1_v5_gn_8_149161.1 | 253773371 | 253774912 | Expressed protein, putative | Identical |
| 55 | Lsat_1_v5_gn_8_149140.1 | 253782079 | 253782273 | Expressed protein, putative | Identical |
| 56 | Lsat_1_v5_gn_8_149100.1 | 253806594 | 253807103 | Auxin-responsive family protein , putative | Identical |
| 57 | Lsat_1_v5_gn_8_148940.1 | 253994600 | 253995929 | CASC3/Barentsz eIF4AIII binding protein, putative | Identical |
| 58 | Lsat_1_v5_gn_8_148920.1 | 254026947 | 254027224 | Expressed protein, putative | Identical |
| 59 | Lsat_1_v5_gn_8_148521.1 | 254100851 | 254101658 | Calcium-binding EF-hand family protein , putative | Identical |
| 60 | Lsat_1_v5_gn_8_148500.1 | 254126447 | 254127221 | Calmodulin-like 38 protein, putative | Identical |
| 61 | Lsat_1_v5_gn_8_148660.1 | 254221012 | 254221455 | Expressed protein, putative | Identical |
| 62 | Lsat_1_v5_gn_8_148641.1 | 254239780 | 254240202 | Calmodulin-like 38 protein, putative | Identical |
| 63 | Lsat_1_v5_gn_8_148600.1 | 254293660 | 254294938 | Expressed protein, putative | Identical |
| 64 | Lsat_1_v5_gn_8_148580.1 | 254347895 | 254348530 | Expressed protein, putative | Identical |
| 65 | Lsat_1_v5_gn_8_148560.1 | 254370689 | 254373701 | Leucine-rich repeat protein kinase family protein , putative | Identical |
| 66 | Lsat_1_v5_gn_8_148540.1 | 254531347 | 254531798 | Expressed protein, putative | Identical |
| 67 | Lsat_1_v5_gn_8_149501.1 | 254729159 | 254731596 | N-terminal nucleophile aminohydrolases (Ntn hydrolases) superfamily protein , putative | Identical |
| 68 | Lsat_1_v5_gn_8_149441.1 | 255003103 | 255003420 | Expressed protein, putative | Identical |
| 69 | Lsat_1_v5_gn_8_149900.1 | 255115797 | 255119868 | Plant U-box 45 protein, putative | Identical |
| 70 | Lsat_1_v5_gn_8_149921.1 | 255173321 | 255174066 | Expressed protein, putative | Identical |
| 71 | Lsat_1_v5_gn_8_150000.1 | 255212128 | 255213957 | Cysteine proteinases superfamily protein , putative | Identical |
| 72 | Lsat_1_v5_gn_8_150021.1 | 255255826 | 255256302 | Expressed protein, putative | Identical |
| 73 | Lsat_1_v5_gn_8_150060.1 | 255313323 | 255313553 | Lumazine-binding family protein , putative | Identical |
| 74 | Lsat_1_v5_gn_8_149561.1 | 255515095 | 255517406 | Ubiquitin-specific protease 12 , putative | Identical |
| 75 | Lsat_1_v5_gn_8_149580.1 | 255562756 | 255566210 | Expressed protein, putative | Identical |
| 76 | Lsat_1_v5_gn_8_149620.1 | 255665392 | 255667651 | Adenylate kinase 1 , putative | Identical |
| 77 | Lsat_1_v5_gn_8_149641.1 | 255667234 | 255671155 | AMP-dependent synthetase and ligase family protein , putative | Identical |
| 78 | Lsat_1_v5_gn_8_149701.1 | 255692392 | 255696157 | Protein kinase superfamily protein , putative | Identical |
| 79 | Lsat_1_v5_gn_8_149721.1 | 255735816 | 255739098 | Expressed protein, putative | Identical |
| 80 | Lsat_1_v5_gn_8_149761.1 | 255774984 | 255778221 | Remorin family protein , putative | Identical |
| 81 | Lsat_1_v5_gn_8_149780.1 | 255806964 | 255807914 | Expressed protein, putative | Identical |
| 82 | Lsat_1_v5_gn_8_149800.1 | 255840058 | 255840480 | Expressed protein, putative | Identical |
| 83 | Lsat_1_v5_gn_8_149841.1 | 256003059 | 256011430 | FAD/NAD(P)-binding oxidoreductase family protein , putative | Identical |
| 84 | Lsat_1_v5_gn_8_150301.1 | 256072859 | 256076087 | Methionyl-tRNA synthetase, putative, putative | Identical |
| 85 | Lsat_1_v5_gn_8_150280.1 | 256078496 | 256087964 | Ribosomal protein L7Ae/L30e/S12e/Gadd45 family protein , putative | Identical |
| 86 | Lsat_1_v5_gn_8_150260.1 | 256109263 | 256113613 | Expressed protein, putative | Identical |
| 87 | Lsat_1_v5_gn_8_150181.1 | 256372487 | 256384015 | DNA-binding storekeeper protein-related , putative | Identical |
| 88 | Lsat_1_v5_gn_8_150161.1 | 256434767 | 256435580 | Calcium-dependent lipid-binding (CaLB domain) family protein , putative | Identical |
| 89 | Lsat_1_v5_gn_8_150141.1 | 256438195 | 256439042 | Expressed protein, putative | Identical |
| 90 | Lsat_1_v5_gn_8_150100.1 | 256457267 | 256460634 | Ankyrin repeat family protein , putative | Identical |
| 91 | Lsat_1_v5_gn_8_151880.1 | 256812209 | 256815553 | Expressed protein, putative | Identical |
| 92 | Lsat_1_v5_gn_8_151840.1 | 256895195 | 256897702 | Tetratricopeptide repeat (TPR)-like superfamily protein , putative | Identical |
| 93 | Lsat_1_v5_gn_8_151781.1 | 256913264 | 256913695 | Calcium-binding EF-hand family protein , putative | Identical |
| 94 | Lsat_1_v5_gn_8_151760.1 | 256916038 | 256918439 | Glyceraldehyde-3-phosphate dehydrogenase C subunit 1 , putative | Identical |
|  |  |  |  |  |  |
